# On the Use of Plasters in Dermatology

**Published:** 1910-12-10

**Authors:** 


### Dermatology.

### ON THE USE OF PLASTERS IN DERMATOLOGY.

The use of plasters has been well established in the practice of medicine since the earliest times, but, curiously enough, almost invariably in the treatment of internal complaints. The emplastrum belladonnæ, for example, has long been a favourite application for the relief of pain in cases of pleurisy and in affections of the joints. In the light of recent research it is doubtful to what extent the benefit which patients ascribe to its use is due to the absorption of belladonna through the unbroken surface of the skin and to what extent the psychical effect of the knowledge that the plaster is sticking firmly to their bodies acts as the true therapeutic influence. In any case good results frequently follow its application. Again mercury has often been administered through the medium of plasters for the treatment of syphilis, especially in France, where the custom was first introduced by the celebrated John of Vigo in the latter half of the fifteenth century. This was a plaster of very abstruse composition, and contained, in addition to mercury, gold, the fat of vipers, and the juice of "quick frogs." In modern preparations these ingredients are omitted. This method of administering mercury is dirty and uncertain, and has now been for the most part abandoned.

The use of plasters, on the other hand, for the treatment of affections of the skin itself has only become prominent in modern times, and the popularisation of this method is largely associated with the name of the distinguished German dermatologist, Professor Unna. He employs plasters which are of two kinds, adhesive and non-adhesive. When non-adhesive plasters are used adhesive plaster is employed also to keep them in position. The old-fashioned lead plaster, which only becomes adhesive after being moistened with turpentine or heated before the fire, is quite unsuitable for application to the skin, for it almost invariably causes a dermatitis. The adhesive plasters which can be used safely are all made up with a solution of indiarubber with which is incorporated a quantity of zinc oxide; such a plaster may be left safely in position for a week or more. A preparation such as this is very useful in any case where it is desirable to obtain protection against external influences such as the friction of the clothes or against scratching. Patches of chronic dermatitis which often are left in the flexures of the limbs after an extensive eczematous eruption has died away from other situations, often do well when protected in this way, and the patients often make the observation that as soon as the plaster is placed in position the itching which they have found so distressing disappears. Many patients with sensitive skin who are liable to small patches of parakeratosis, particularly where the surface is subject to a little additional friction—for example, where the buckle of the sock-suspender rubs slightly, or after carrying a gun upon the shoulder—find that these patches disappear in a surprisingly short time if the place affected be protected with a piece of zinc oxide plaster of suitable size. It is also very useful in cases of small patches of lichen planus, in which the irritation is ofttimes intense. These small lesions if protected by plaster cease to itch at once. As they do not itch they are not scratched, and as it is the scratching which is the chief cause of their persistence and spread they speedily vanish away. In a generalised eruption of lichen planus it is, of course, impossible to cover all the papules with a coating of plaster, but in these cases it often happens that there is more irritation in one situation than another, and the protection of one or even several small areas will frequently give great relief. Another condition in which zinc oxide plaster is distinctly useful is when the skin has been grazed so that the epidermis has been rubbed off and thus the papillæ laid bare—the sort of wound which is produced by knocking the hand against a hard and rough surface. It will be found that these annoying little lesions heal over much more quickly when protected by a coating of zinc plaster than under any other conditions.

Other varieties of plaster are also made up containing many different drugs—for example, mercury, salicylic acid, and resorcin are frequent constituents. Mercurial plasters, although unsuitable as a medium for the internal administration of mercury, are useful for promoting the absorption of syphilitic deposits when locally applied, and may be more particularly recommended when the hands or face are affected and the lesions are unpleasantly conspicuous. They are also useful for application to boils and furuncles on the back of the neck or on the limbs. Salicylic acid is a very useful drug when made up in the form of a plaster, owing to its power of reducing or softening hard and horny structures. Sometimes it is used for ordinary warts, but as a rule there are more convenient methods of dealing with these troublesome disfigurements. On the other hand, salicylic plasters containing about 25 per cent. of the acid form perhaps the best application for the multiple papillomata which not uncommonly occur upon the heel and sole of the foot, and which usually, on account of their exquisite tenderness, make walking an extremely painful process. Under the influence of a salicylic plaster these soften, and if they do not actually fall out can easily be removed with a corn-knife. Nor do they often recur. A plaster is also a convenient way of applying salicylic acid in cases where hyperkeratosis complicates either eczematous or syphilitic lesions of the palms or soles. Reduction of the very conspicuous horny covering which so often is present is a most necessary condition of the successful treatment of these conditions. Another type of case wherein the same sort of plaster can be recommended is lupus vulgaris. The application of a strong preparation of salicylic acid causes the nodules to soften and ulcerate, and if the application be repeated sufficiently often it may indeed alone effect a cure—but the process is slow. A speedier method is to apply the plaster until the patch is quite sore, and it will always be found that the lupus nodules are more affected than the healthy portions of the skin, and then to submit the whole to $x$-rays. Good results can often be obtained by this combination of therapeutic agents, both from a radical and a cosmetic standpoint, and the time required is a mere fraction of that occupied by a course of Finsen light treatment.

This treatment is therefore particularly to be recommended in old-standing and extensive cases. An excellent and permanent scar can be obtained if the case be well managed.

